# Retrieval of the Aveir™ leadless pacemaker with the double-snare technique

**DOI:** 10.1093/ehjcr/ytae267

**Published:** 2024-05-30

**Authors:** Junji Morita, Yusuke Kondo, Yuhei Kasai, Takayuki Kitai

**Affiliations:** Department of Cardiovascular Medicine, Sapporo Cardiovascular Clinic, North 49, East 16, 8-1, Higashi Ward, Sapporo, Hokkaido 007-0849, Japan; Department of Cardiovascular Medicine, Chiba University Graduate School of Medicine, 1-8-1 Inohana, Chuo-ku, Chiba-shi, Chiba 260-8670, Japan; Department of Cardiovascular Medicine, Sapporo Cardiovascular Clinic, North 49, East 16, 8-1, Higashi Ward, Sapporo, Hokkaido 007-0849, Japan; Department of Cardiovascular Medicine, Sapporo Cardiovascular Clinic, North 49, East 16, 8-1, Higashi Ward, Sapporo, Hokkaido 007-0849, Japan

An 84-year-old man underwent Aveir™ leadless pacemaker (LP) implantation for bradycardia with atrial fibrillation. In this case, the decision to opt for a LP was influenced by the patient’s advanced age, dementia, and chronic kidney disease (stage 4), which increased the risk of pocket infections. During the procedure, the current of injury was poor, the pacing impedance was 280 Ω, the pacing threshold was 0.5 V/0.4 ms, and the sensing amplitude was 6.5 mV (*Figure 1A*) at the implantation site. We reassessed the data after a brief period in tether mode; all parameters remained consistent with the initial assessment. The LP remained fixed during the deflection test (*Figure 1B*) and was released at that site. The tether did not immediately detach from the LP (*Figure 1C*); however, the LP became dislodged from the myocardium (*Figure 1D*) and the tether detached from the LP (see Supplementary material online, *Movie S1*, *Figure 1E*). The dislodged LP migrated from the right ventricle and floated within the right atrium. To prevent the LP from migrating to the pulmonary artery, we inserted a snare catheter (Osypka Medical GmBH, Berlin, Germany) and an 8.5 Fr steerable sheath (Agilis NXT; St. Jude Medical, St Paul, MN, USA) to grasp the main body, and we caught the docking button with the tri-loop snare of the Aveir retrieval catheter to remove the LP (see Supplementary material online, *Movie S2*, *Figure 1F*). After retrieval, we examined the catheter system and consulted the manufacturer; no defects or malfunctions were identified. There are several reports of dislodged LP retrieval, but most are related to the Medtronic Micra™.^1,2^ There have been few reports of Aveir™ retrieval using double snares.^3^ Even if the LP passes the deflection test, it is important to check for dislodgement when the current of injury is low.

**Figure 1:**
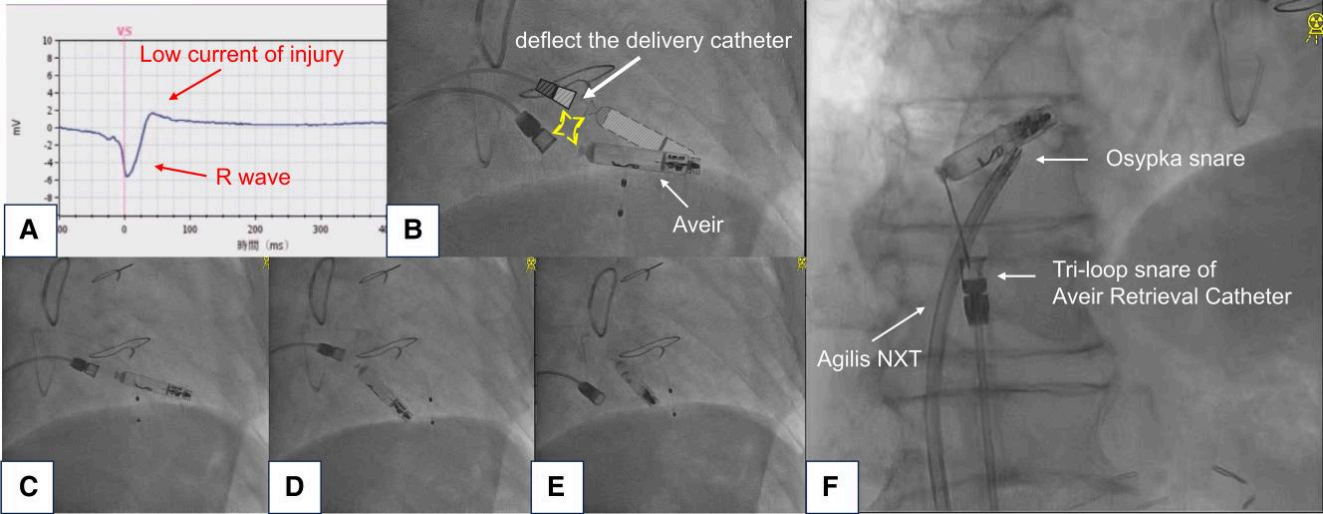
(*A*) EGM following screwing of the leadless pacemaker, indicating a low current of injury. (*B*) Illustration of the deflection test to confirm leadless pacemaker fixation by delivery catheter deflection. (*C*–*E*) Leadless pacemaker dislodgement. (*F*) Double-snare technique.

## Supplementary Material

ytae267_Supplementary_Data

## Data Availability

The data underlying this article will be shared upon reasonable request to the corresponding author.

## Supplementary material

**Consent:** The authors confirm that written consent for submission and publication of this case report including image and associated text has been obtained from the patient in line with COPE guidance.

**Funding:** None declared.
